# Beyond urodynamics: non-invasive approaches to diagnosing detrusor underactivity in men with lower urinary tract symptoms – a systematic review

**DOI:** 10.1186/s12894-025-01722-w

**Published:** 2025-03-06

**Authors:** Karolina Garbas, Łukasz Zapała, Aleksander Ślusarczyk, Hanna Piekarczyk, Tomasz Piecha, Piotr Radziszewski

**Affiliations:** Department of General, Oncological and Functional Urology, Medical University of Warsaw, Lindleya 4, 02-005 Warsaw, Poland

**Keywords:** Detrusor underactivity, Non-invasive tests, Underactive bladder, Urodynamics, Pressure flow study, LUTS

## Abstract

**Background:**

To evaluate and synthesize existing evidence on non-invasive methods for diagnosing detrusor underactivity (DU) in men presenting with lower urinary tract symptoms (LUTS), focusing on their feasibility and diagnostic accuracy.

**Methods:**

A systematic search of PubMed, Scopus, and Web of Science databases was conducted for original articles reporting on non-invasive diagnostic tests for DU in men with LUTS. Data extraction focuses on study characteristics, diagnostic methods, and accuracy. The risk of bias was assessed using the QUADAS-2 tool.

**Results:**

Eighteen studies involving 7390 patients, of whom 3194 were diagnosed with DU, were included in our analysis. The evaluated diagnostic methods included ultrasound parameters, biomarkers, uroflowmetry results, symptom questionnaires, and clinical characteristics. Developed models, including those based on artificial intelligence (AI), and nomograms were also assessed. The symptom questionnaire DUA-SQ showed the highest sensitivity of 95.8%, while ultrasound measurements, such as detrusor wall thickness showed 100% specificity but limited sensitivity (42%). Models incorporating clinical variables achieved sensitivity rates of over 75%. Uroflowmetry parameters, particularly presence of "sawtooth" and "interrupted" waveforms, demonstrated sensitivity of 80% and specificity of 87%. Biomarkers, including serum adiponectin and urine NO/ATP ratio, achieved sensitivity of 79% and 88.5%, respectively. AI models showed potential, with sensitivities ranging from 65.9% to 79.7%. Due to the poor quality of the studies and data heterogeneity, meta-analysis was not performed.

**Conclusions:**

Non-invasive diagnostic methods for DU, particularly DUA-SQ, ultrasound measurements, and AI models, demonstrate potential, though their accuracies vary. Further research is needed to standardize these methods and enhance their diagnostic reliability.

**Trial registration:**

The study protocol was registered with PROSPERO (CRD42024556425). Clinical trial number: not applicable.

## Background

Detrusor underactivity (DU) has recently gained attention as a significant phenomenon among patients with lower urinary tract symptoms (LUTS). However, despite its growing recognition, the medical condition still lacks a standardized definition and universally accepted diagnostic criteria [1]. The International Continence Society (ICS) defined DU as *“a contraction of reduced strength and/or duration, resulting in prolonged bladder emptying and/or failure to achieve complete bladder emptying within a normal time span”* [2]. Recently, D’Ancona et al. revised this definition and described DU as *“low detrusor pressure or short detrusor contraction time, usually in combination with a low urine flow rate resulting in prolonged bladder emptying and/or a failure to achieve complete bladder emptying within a normal time span”* [3].

A separate term, “underactive bladder” (UAB), has been introduced to describe the symptoms associated with DU [4]. Nevertheless, defining UAB is challenging due to its lack of specific symptoms and frequent overlap with those of overactive bladder (OAB) and bladder outlet obstruction (BOO), which makes the diagnosis especially challenging [5]. DU can, thus, present as a wide array of LUTS, including urgency, frequency, and nocturia in the storage phase, as well as hesitancy, weak or interrupted stream, straining to void, and a feeling of incomplete emptying during the voiding phase [6].

The prevalence of DU is estimated to range from 9 to 23% in men under 50 years old, increasing to approximately 48% in men over 70 years old. In older women, the prevalence is estimated to be between 12 and 45% [6]. However, accurately determining prevalence remains difficult due to the absence of widely accepted non-invasive diagnostic methods [5].

The current gold-standard for diagnosing DU, proposed by the ICS, is invasive urodynamics (UDS), which includes cystometry followed by a pressure-flow study (PFS) [7]. However, for an extended period of time, the ICS did not specify exact thresholds for diagnosing DU or define a normal bladder emptying time, leading to unstandardized diagnostic criteria [8]. One of the most commonly used criteria for men in both clinical practice and research is the Bladder Contractility Index (BCI), introduced by Abrams et al. in 1999 [9]. The BCI is calculated using the formula *BCI* = *Pdet@Qmax* + *5Qmax*, which in turn provides a quick and easily applicable assessment of detrusor contractility. A BCI below 100 suggests DU, a BCI between 100 and 150 is considered normal, and a BCI greater than 150 indicates the presence of strong detrusor [9].

In 2023, the ICS and the Society of Urodynamics, Female Pelvic Medicine & Urogenital Reconstruction (SUFU) recommended adopting the BCI under the new term “Detrusor Contraction Index” (ICS-DCI), retaining the same formula for use in clinical practice, scientific analyses and cohort reporting [10]. The working group replaced the terms “contractility” with “contraction” and “bladder” with “detrusor,” and advised using ICS-DCI as a continuous scale to grade detrusor voiding contraction (DVC). They suggested the clinical classes “normal DVC” and “weak DVC”, while also recognizing “strong DVC” and “very weak DVC” for specific sub-cohorts (male patients). Although the group proposed a nomogram (the ICS-PFS plot nomogram) with a recommended cutoff at DCI = 100 to differentiate normal from weak DVC, it also called for further studies on the provisional cutoffs of DCI = 50 (very weak) and DCI = 150 (strong) [10].

In addition to the lack of standardized diagnostic criteria, invasive PFS are usually time-consuming, and may pose a risk of infectious complications in predisposed individuals, while at the same time are not widely accessible due to relatively high costs and the need for highly trained urodynamics specialists. Thus, patients are often reluctant to undergo these invasive procedures because of the associated discomfort and above-mentioned potential risk of infection. Consequently, there has been a growing interest in developing new non-invasive diagnostic methods, including uroflowmetry (UFL) analysis [11, 12], bladder wall thickness (BWT) or detrusor muscle thickness (DMT) measurement via ultrasound [13, 14], use of artificial intelligence (AI) [15, 16] and potential biomarkers in blood serum [17] or urine [18].

Consequently, the aim of our study was to assess the diagnostic value and feasibility of emerging non-invasive tests for diagnosing DU in men with LUTS compared to the invasive gold-standard PFS.

## Methods

### Search strategy

Our study protocol was registered with PROSPERO (CRD42024556425).

A systematic literature review was performed in accordance with the Preferred Reporting Items for Systematic Reviews and Meta-analyses (PRISMA) statement to identify all scientific studies investigating a non-invasive diagnostic method for DU in men with non-neurogenic LUTS.

The Population, Intervention, Control, Outcome (PICO) framework was used to specify the review question. The following inclusion criteria were considered for evaluation:

- Type of study and study population (P): We included only full-text original studies in English language, published after the year 2000, covering a non-invasive diagnostic method for DU. Non-invasive method meant no need for performing an invasive PFS. The study population consisted of adult men (≥ 18 years old) with non-neurogenic LUTS. We excluded studies that included females in the study group or patients with neurogenic bladder or any diagnosed neurologic disease that could account for neurogenic LUTS.
- Index test (I): Index tests included any type of non-invasive tests or predictors used for the diagnosis of DU.
- Reference standard (C): In all studies, the reference standard used for the diagnosis of DU must have included PFS (conventional, video, or ambulatory urodynamics).
- Outcome (O): The accuracy of the non-invasive methods was reported using sensitivity, specificity, positive predictive value (PPV), negative predictive value (NPV) and overall accuracy for the diagnosis of DU.

We searched Medline (PubMed), Scopus and Web of Science databases in June 2024 for all relevant publications to date using the following terms: (“underactive bladder” OR “detrusor underactivity” OR “impaired detrusor contractility” OR “acontractile detrusor” OR “underactive detrusor” OR “detrusor failure” OR “detrusor areflexia”).

### Data selection

**Fig. 1:**
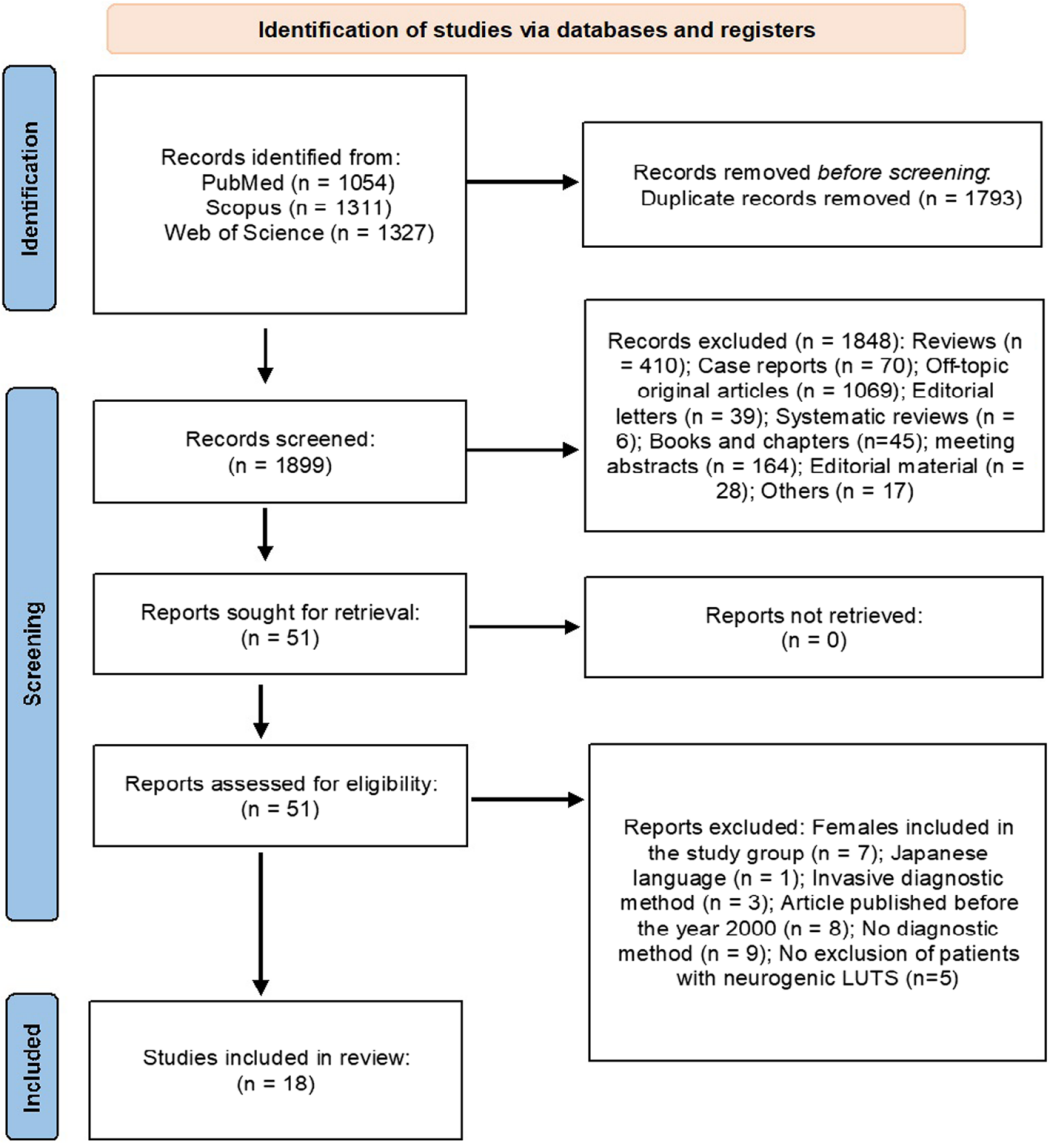
Flowchart for study selection process using Medline, Web of Science and Scopus databases to identify the original articles investigating a non-invasive diagnostic method for DU in men with non-neurogenic LUTS

Abstracts of all identified studies after exclusion of duplicates (*n* = 1899, see Fig. 1 for details) were independently reviewed and screened for eligibility by two authors (KG, ŁZ). Further selected original studies reporting on non-invasive diagnostic methods for DU were reviewed in full text (*n* = 51). Disagreements between individual judgements, when present, were referred to the third reviewer (TP), and the final decision was made based on the majority vote of the three reviewers. Finally, we decided to include 18 studies in our review. Studies were only included in our systematic review, if they were original studies in English language with full-text available and they covered any type of non-invasive tests used for the diagnosis of DU or DU predictors in men with non-neurogenic LUTS. Data from the eligible reports and their supplementary data files were extracted by two reviewers independently (KG, HP). Any discrepancies were resolved through consensus among the authors.

Studies that utilized invasive methods, specifically those diagnosing DU through UDS, were excluded [19–21]. Nevertheless, we opted to include two studies [13, 14] that involved cystometry to standardize the level of bladder filling. In these studies, the DMT/BWT ratio and BWT were measured with the use of ultrasound. Our rationale for including these studies was that, despite the invasive nature of cystometry used for standardizing the measurement conditions, the method itself employed ultrasound, which could be used non-invasively in clinical practice. Patients could achieve an appropriate bladder filling by drinking a precise amount of fluids before the test. Studies in which the exclusion criteria did not precisely mention neurogenic bladder or a diagnosed neurogenic disease were excluded [22–26]. We also excluded studies performed before the year 2000, as we found the data outdated in comparison to the most up-to-date knowledge about DU.

### Data display

All data extracted from the studies were systematically organized and presented using tables to ensure clarity and facilitate comparison across studies. Descriptive statistics, including means, medians, percentages, and ranges were displayed where applicable. When presenting the accuracy of the examined methods, sensitivity, specificity and prevalence were used to calculate missing NPV and PPV values, where available.

### Risk of bias assessment

We used the Quality Assessment of Diagnostic Accuracy Studies 2 (QUADAS-2) Critical Appraisal Tool to evaluate the methodological quality and risk of bias in the retrieved studies [27]. Two authors (KG and HP) individually and independently assessed all the studies based on the QUADAS-2 criteria, and any potential disagreements were resolved via consensus between those two authors. In the index test domain, the risk of bias was deemed “low” when the physicians interpreting the test were blinded to the reference standard results, and when the measurement methods were consistent across all patients (e.g., all measurements were performed using abdominal ultrasound). For the reference standard domain, the risk of bias was deemed “low” if the PFS was conducted according to ICS Standards by a physician blinded to the index test results, if both DU and non-DU patients underwent the reference standard, and if the diagnosis of DU was based on BCI and BOOI values. The risk of bias in the flow and timing domain was considered “low” when patient flow was clearly documented, all patients included in the study were included in the analysis, and all received the same reference standard. Regarding applicability concerns, most studies were assessed as having “low” concerns, with some exceptions. The reference standard domain was classified as “unclear” when diagnostic criteria other than BCI/BOOI were used to diagnose DU. The QUADAS-2 template for tabular display was employed to visualize the risk of bias and applicability [28] and the Robvis tool was utilized to create a summary plot of the risk of bias of all included studies [29].

## Results

### Characteristics of included studies

This systematic review included 18 studies with a total of 7390 patients, of whom 3194 were diagnosed with DU.

The review comprised 18 observational studies, including cross-sectional studies (*n* = 17) and a case–control study (*n* = 1). The studies were conducted across multiple geographic regions, with a majority from Asia (*n* = 13), followed by Europe (*n* = 3), Turkey (*n* = 1) and India (*n* = 1). The publication dates ranged from 2016 to 2024 (see Table 1 for details).

**Table 1:** Characteristics of the studies included in the systematic review

No	Study	DOI	Country	Study design	Dates of analysed data	Inclusion criteria	Exclusion criteria
1	Kiba et al. 2022 [ 30]	10.1111/luts.12424	Japan	retrospective cross-sectional study	January 2013—October 2020	men ≥ 50 years without neurogenic disorders with DU or BOO	neither DU nor BOO, surgery for BPH, conditions known to cause neurogenic bladder dysfunction (neurological disease, spinal disease, cerebrovascular infarction, or cerebral hemorrhage), PCa, BCa, incomplete data
2	De Nunzio et al. 2020 [14]	10.1002/nau.24327	Italy	prospectively recruited, retrospective cross-sectional study	January 1996—December 2000	males, ≥ 45 years old, LUTS, informed consent	neurological disorders, renal insufficiency, bladder stones, PCa, urethral stricture, previous pelvic surgery, taking α‐blockers and 5‐α reductase inhibitors
3	Matsukawa et al. 2020 [11]	10.1111/iju.14121	Japan	prospectively recruited, retrospective cross-sectional study	2010–2017	male patients, total IPSS ≥ 8, IPSS-QOL ≥ 3, PV ≥ 20 mL in transabdominal US and age ≥ 45 years	pharmacotherapy for LUTS, bladder calculi, active UTI, suspected of conditions known to cause neurogenic bladder dysfunction (severe diabetes mellitus, neurological disease, history of pelvic surgery, spinal disease, cerebrovascular infarction or cerebral hemorrhage), incomplete data
4	Rademakers et al. (FORCE Research Group) 2017 [31]	10.1007/s00345-016–1902-7	the Netherlands, Germany	prospectively recruited, retrospective cross-sectional study	1 January 2000—31 December 2001	treatment-naïve men ≥ 40 years with clinical BPH, LUTS, and/or PV > 25 ml and able to void without previous medical or surgical therapies for LUTS	α-blockers, 5α-reductase inhibitors, urinary retention, prior LUT or pelvic surgery, evident PCa or BCa, or a neurologic deficit
5	Matsukawa et al. 2021 [16]	10.1111/iju.14661	Japan	retrospective cross-sectional study	January 2015 to December 2018	male patients, total IPSS of ≥ 8, IPSS-QOL of ≥ 3 and age of ≥ 45 years	prior LUTS pharmacotherapy, bladder calculi, active UTI or suspected neurogenic bladder dysfunction due to severe DM, neurological disease, history of pelvic surgery, spinal disease, cerebrovascular infarction or cerebral hemorrhage
6	Takahashi et al. 2021 [32]	10.1002/nau.24558	Japan	retrospective cross-sectional study	January 2010—January 2017	male LUTS patients who underwent PFS	patients < 50 years, duplicates, with history of BPH surgery, neurogenic bladder, PCa, IC, BCa, bladder stones, and others affecting LUT, without full PFS data because of urinary retention or minimal VV (< 50 ml), without the data: IPSS, UFL, PVR, and PV; with DO on PFS
7	Namitome et al. 2020 [33]	10.1097/JU.0000000000000616	Japan	retrospective cross-sectional study	January 2010—January 2017	male patients with LUTS, ≥ 50 years old	previous BPH surgery, neurogenic bladder, PCa, IC, BCa, bladder stones, bladder neck contracture, chronic prostatitis, urethral stricture, urinary retention, < 50 ml on PFS, no full data
8	Matsukawa et al. 2023 [34]	10.1080/13685538.2018.1481941	Japan	retrospective cross-sectional study	September 2018—December 2021	men with non-neurogenic LUTS who underwent UFL and PFS	total voided and residual urine volumes in UFL < 100 mL
9	Kim et al. 2019 [ 35]	10.1080/13685538.2018.1481941	Korea	retrospective cross-sectional study	2000 – 2016	male patients who underwent UDS	DO in UDS, acute urinary retention, neurogenic problems including stroke, Parkinson’s disease, spinal cord injury, and acute UTI; low BCI and a high BOOI, suggesting simultaneous DU and BOO
10	Ishikawa et al. 2023 [17]	10.1007/s00345-023–04341-y	Japan	prospective cross-sectional study	April 2018—December 2020	treatment-naïve men aged ≥ 65 years with non-neurogenic LUTS	oral treatment for LUTS, neurogenic bladder dysfunction, bladder calculi, active UTI; severe cardiac disease (NYHA ≥ grade 2), hepatic dysfunction (AST and ALT levels > 2 × the normal values); renal dysfunction (serum creatinine ≥ 2.0 mg/dL); treatment for cancer at the time of study entry, receiving thiazolidinedione derivatives for DM
11	Krishnan et al. 2021 [18]	10.1007/s00345-021–03784-5	India	prospective case–control study	June 2017—November 2018	cases: male patients aged 18–70 years with LUTS and PVR of > 150 ml; controls: healthy age- and sex-matched volunteers without any LUTS (zero IPSS Score)	cases: neurogenic bladder dysfunction, medications affecting bladder contractility such as anticholinergics, antipsychotics, opioids or beta-3 agonists; controls: serum creatinine > 2 mg/dl, prolonged use of urethral catheter, active UTI
12	Luo et al. 2017 [36]	10.1007/s11255-017–1539-5	China	retrospective cross-sectional study	January 2013—June 2016	adult men with BPH/LUTS	neurogenic bladder with a confirmed etiology, such as pelvic trauma; previous medical or surgical therapies for BPH/LUTS
13	Lee et al. 2022 [13]	10.1038/s41598-022–09302-w	Korea	prospectively recruited, retrospective cross-sectional study	December 2017—October 2019	male patients ≥ 40 years scheduled for UDS due to LUTS/BPH refractory to medical treatments for > 3 months	medical conditions affecting bladder function and/or structural deformation including neurological disorders of parkinsonism, stroke, MS, urethral stricture, bladder diverticulum, bladder stone, previous BPH or pelvic surgery, and PCa; patients with complications of Clavien–Dindo grading 3 or higher after prostate surgery
14	Bang et al. 2022 [15]	10.4111/icu.20210434	Korea	retrospective cross-sectional study	December 2006—Decemer 2017	males, ≥ 40 years old, who underwent PFS	diseases affecting LUT function, BCa, PCa, previous prostate, bladder, and/or urethral surgeries, indwelling catheters (or needing regular catheterization), history of cerebrovascular accident, neurologic disorders, and spinal or pelvic bone trauma, < 150 mL voided on UFL, insufficient UFL study graphs for analysis
15	Lee et al. 2016 [12]	10.4111/icu.2016.57.6.437	Korea	retrospective cross-sectional study	2008—2014	men > 50 years old who visited outpatient department complaining of weak stream, hesitation, intermittent urination and residual urine sense, with IPPS ≥ 8 points, normal serum PSA < 3.5 ng/dL, no hematuria or pyuria and no α-blocker, anticholinergics or beta-agonists for min. 8 weeks before evaluation	central or peripheral neurogenic disease including cerebral vascular accident and spinal cord disease, histories of urinary tract abnormalities or lithiasis, surgeries of the pelvic floor or bladder, chronic pelvic pain, cardiovascular disease, patients unable to complete UDS, other UDS criteria than: DU: BCI < 100 cmH2O with BOOI; < 20 cmH2O, and BOO: BOOI ≥ 40 cmH2O
16	Yoldas 2022 [37]	10.4081/aiua.2022.1.51	Turkey	retrospective cross-sectional study	January 2007—January 2015	males with min. 2 UFLs (and ≥ 150 ml on UFL) and PVR measurements	females, urological malignancies, calculi in the bladder and lower end of the ureter, infection and asymptomatic bacteriuria, transurethral intervention history, neurogenic disease, indwelling catheter or CIC, bedridden patients, missing data
17	Wu et al. 2024 [38]	10.22514/jomh.2024.031	China	retrospective cross-sectional study	January 2018—October 2023	BPH/LUTS patients, informed consent form, 18—100 years old; conscious, compliant, ability to express feelings and independently complete symptom questionnaire; diagnosis made through physical examinations, PV measurement by abdominal US and UDS; DU defined with the PIP < 100	PV > 50 mL; history of prostate surgery, prostate biopsy, medication (antimuscarinic, beta3 agonists, α1-antagonists, 5α-reductase inhibitor, intravesical injection of Botulinum Neurotoxin A and hyaluronic acid), acute and chronic prostatitis; urethral stricture, radical pelvic surgery, neurologic disease, OAB, urinary incontinence, DM and no other periferic neurologic condition to explain DU (Tarlov cyst, severe lumbar hernia, pelvic trauma)
18	Garbas et al. 2024 [39]	10.1007/s11255-024–04093-7	Poland	retrospective cross-sectional study	2012 – 2022	male gender, age > 18 years old, LUTS, final diagnosis of DU or BOO in PFS, UFL performed immediately prior to undergoing PFS, VV on UFL > 150 ml, informed consent	neurogenic bladder (any history of neurologic disease), chronic prostatitis, IC, BCa, PCa, bladder stones, prior BPH surgery, incomplete data

*BCI* bladder contractility index, *BCa* bladder cancer, *BOO* bladder outlet obstruction, *BOOI* bladder outlet obstruction index, *BPH* benign prostatic hyperplasia, *CIC* Clean Intermittent Catheterization, *DM* diabetes mellitus, *DO* detrusor overactivity, *DU* detrusor underactivity, *IC* Interstitial cystitis, *IPSS* International Prostate Symptom Score, *IPSS-QOL* International Prostate Symptom Score Quality of Life, *LUT* lower urinary tract, *LUTS* lower urinary tract symptoms, *MS* multiple sclerosis, *OAB* overactive bladder, *PCa* prostate cancer, *PFS* pressure flow study, *PIP* projected isovolumetric pressure, *PSA* prostate specific antigen, *PV* prostate volume, *PVR* post-void residual, *UDS* urodynamic study, *UFL* uroflowmetry, *US* ultrasound, *UTI* urinary tract infection

Sample sizes varied from 44 to 1792 participants, with a median sample size of 252. All studies exclusively involved male patients, with a mean or median age over 50 years. The studies addressed diverse populations, including study groups with pure DU (*n* = 17) and comparator groups consisting of individuals without DU (*n* = 11), DU combined with BOO (*n* = 1), BOO alone (*n* = 4), or healthy controls (*n* = 1). One study included two separate analyses: one compared a DU group with a DU + BOO + and BOO group, while the other compared DU + BOO + to both DU and BOO groups [16]. Baseline characteristics of patients are presented in Table 2.

**Table 2:** Baseline characteristics of patients included in the studies. Values are presented as mean +—SD, unless indicated as median (Q1-Q3)

No	Study	Sample size (n)	DU group	Comparator group	DU (n)	Comparator (n)	Age (DU)	Age (comparator)	Medication (DU)	Medication (comparator)	Comorbidities (DU)	Comorbidities (comparator)
1	Kiba et al. 2022 [30]	205	pure DU	DU + BOO + , BOO	63	DU + BOO + 48; pure BOO 94	73.9 + -6.7	DU + BOO + 71.2 ± 7.1; pure BOO 71.6 ± 6.4	α-1 blocker 47 (75%); Tadalafil 17 (27%); Dutasteride 16 (25%); Anticholinergic agent or beta-3 adrenergic agonist 5 (8%)	DU + BOO + : α-1 blocker 42 (86%); Tadalafil 7 (15%); Dutasteride 25 (52%); Anticholinergic agent orbeta-3 adrenergic agonist 5 (10%); BOO + : α-1 blocker 82 (87%), Tadalafil 18 (19%), Dutasteride 42 (45%), Anticholinergic agent or beta-3 adrenergic agonist 18 (19%)	DM: 7 (11%)	DU + BOO + : DM- 5 (10%), pure BOO: DM- 15 (16%)
2	De Nunzio et al. 2020 [14]	448	DU	non-DU	194	254	67.6 ± 7.5	67.8 ± 8.1	no α‐blockers and 5‐α reductase inhibitors	no α‐blockers and 5‐α reductase inhibitors		
3	Matsukawa et al. 2020 [11]	418	DU	BOO	145	273	69.5 + -9.9	69.7 + -7.1	no LUTS pharmacotherapy	no LUTS pharmacotherapy		
4	Rademakers et al. (FORCE Research Group) 2017 [31]	143	DU	non-DU	33	110	median 62 (59–73)	median 62 (57–68)	no LUTS pharmacotherapy	no LUTS pharmacotherapy		
5	Matsukawa et al. 2021 [16]	264	DU	BOO, DU + BOO +	81	183	70.1 + -8.8	BOO 68.9 + -7.2; DU + BOO 71.4 + -8.3	no LUTS pharmacotherapy	no LUTS pharmacotherapy		
	Matsukawa et al. 2021 [16]		DU + BOO +	DU, BOO	65	199	71.4 + -8.3	BOO 68.9 + -7.2; DU 70.1 + -8.8	no LUTS pharmacotherapy	no LUTS pharmacotherapy		
6	Takahashi et al. 20210 [32]	909	DU	non-DU	454	455	71.7 + -8.6	69.4 + -7.6				
7	Namitome et al. 2020 [33]	909	DU	non-DU	454	455			of the whole cohort: some patients with LUTS refractory to medication	of the whole cohort: some patients with LUTS refractory to medication		
8	Matsukawa et al. 2023 [34]	266	DU	non-DU (BOO group, DU + BOO group, and non-DU + non-BOO group)	70	196	69.8 + -8.7	70.2 + -7.9				
9	Kim et al. 2019 [35]	318	pure DU	pure BOO	165	153	67 + -8.8	65 + -15.1				
10	Ishikawa et al. 2023 [17]	118	DU	non-DU	39	79	median 76 (68–81)	median 76 (68–81)	no α1-blockers, phosphodiesterase type 5 inhibitors, 5α-reductase inhibitors, anticholinergic agents, β3-adrenergic receptor agonists or thiazolidinedione derivatives for DM	no α1-blockers, phosphodiesterase type 5 inhibitors, 5α-reductase inhibitors, anticholinergic agents, β3-adrenergic receptor agonists or thiazolidinedione derivatives for DM	HTN: 27, HL: 28, DM: 7	HTN: 51, HL: 49, DM: 21
11	Krishnan et al. 2021 [18]	44	DU	healthy controls	26	18	56.7 + -16.7	52.3 + -14.9	no medications affecting bladder contractility (anticholinergics, antipsychotics, opioids or beta-3 agonists)		DM: 4, BOO: 8	
12	Luo et al. 2017 [36]	704	DU	non-DU	112	592	70.3 + -8.4	69.6 + -7.8	no LUTS pharmacotherapy	no LUTS pharmacotherapy	HTN: 53, DM: 11, MI: 21, stroke: 19	HTN: 230, DM: 100, MI: 120, stroke: 99
13	Lee et al. 2022 [13]	94	DU	non-DU	24	70	median 73 (70.5–75.5)	median 71 (66–74)	LUTS treatment > 3 months	LUTS treatment > 3 months		
14	Bang et al. 2022 [15]	1792	pure DU	non-DU	893	899	64.93	64.39				
15	Lee et al. 2016 [12]	240	DU	BOO	111	129	68.01 + -9.74	68.39 + -9.29	no LUTS pharmacotherapy for min. 8 weeks prior to UDS	no LUTS pharmacotherapy for min. 8 weeks prior to UDS		
16	Yoldas 2022 [37]	93	pure DU	pure BOO	44	49	78.54 + -11.6	64.18 + -11.1	drugs affecting LUTS stopped 3 days prior to UDS	drugs affecting LUTS stopped 3 days prior to UDS		
17	Wu et al. 2024 [38]	196	DU	non-DU	93	103	70.7 ± 6.9	67.6 ± 7.4	no antimuscarinic, beta3 agonists, α1-antagonists, 5α-reductase inhibitor	no antimuscarinic, beta3 agonists, α1-antagonists, 5α-reductase inhibitor	HTN: 38, DM: 12, Cardiovascular Disease: 19, Bladder stone: 10, Hyperlipidemia: 38	HTN: 32, DM: 10, Cardiovascular Disease: 13, Bladder stone: 14, Hyperlipidemia: 40
18	Garbas et al. 2024 [39]	229	DU	non-DU	128	101	median 61.5 (49–69.5)	median 63 (52–70)	cholinolitycs: 17, α-blockers: 36, insulin: 3, oral drugs for DM: 4, statins: 6	cholinolitycs: 6, α-blockers: 52, insulin: 3, oral drugs for DM: 12, statins: 4	DM: 12, hypothyroidism: 6	DM: 16, hypothyroidism: 5

*BOO* bladder outlet obstruction, *DM* diabetes mellitus, *DU* detrusor underactivity, *HL* hyperlipidemia, *HTN* hypertension, *LUTS* lower urinary tract symptoms, *MI* myocardial infarction, *UDS* urodynamic study

BCI data were available in 11 studies for both DU and comparator groups and in one study for the DU group only. BOOI data were available in 13 studies. Other UDS data were less commonly reported, with Pdet@Qmax available in 9 studies, Qmax in both DU and comparator groups in 8 studies, and in 1 study for the DU group only. Pdetmax data were reported in only 2 studies (see Table 3 for details).

**Table 3:** UDS characteristics of the patients included in the studies. Values are presented as mean +—SD, unless indicated as median (Q1-Q3)

No	Study	UDS criteria for DU diagnosis	UDS criteria for comparator group diagnosis	Pdet@Qmax (DU group)	Pdet@Qmax (comparatorgroup)	Qmax_ PFS(DU group)	Qmax_PFS (comparator group)	Pdetmax (DU group)	Pdetmax (comparator group)	BCI (DU group)	BCI (comparator group)	BOOI (DU group)	BOOI (comparator group)
1	Kiba et al. 2022 [30]	BCI < 100 and BOOI < 40	DU + BOO group: BCI < 100 and BOOI ≥ 40; pure BOO group: BCI ≥ 100 and BOOI ≥ 40	35.3 ± 12.1	DU + BOO + : 66.1 ± 12.3, pure BOO: 98.3 ± 25.4	7.0 ± 2.7	DU + BOO + 4.1 ± 1.8; pure BOO: 5.4 ± 2.5			70.4 ± 19.5	DU + BOO + : 86.5 ± 9.7; pure BOO: 125.4 ± 22.5	21.2 ± 12.4	DU + BOO + : 57.9 ± 14.8, pure BOO: 87.5 ± 28.1
2	De Nunzio et al. 2020 [14]	BCI < 100	BCI > 100	42 ± 16	67 ± 31	6.1 ± 3.1	9.2 ± 5.2			78.8 ± 13.6	130 ± 25	28 ± 20	43 ± 40
3	Matsukawa et al. 2020 [11]	BCI ≤ 100 and BOOI ≤ 40	BCI 100 and BOOI > 40							73.1 ± 19.0	127.4 ± 20.6	26.8 ± 9.2	73.2 ± 25.8
4	Rademakers et al. (FORCE Research Group) 2017 [31]	by exclusion: patients with PVR > 30 ml but without BOO or dysfunctional voiding in PFS		median 27.2 (19.8–39.8)	median 58.2 (45.6–76.6)							median 15.6 (5.2–27.2)	median 39.2 (22.9–63.1)
5	Matsukawa et al. 2021 [16]	BCI ≤ 100 and BOOI ≤ 40	BOO: BCI > 100 and BOOI > 40							DU: 68.8 ± 18.3	BOO: 121 ± 22; BOO + DU: 84.9 ± 9.1	DU: 27.1 ± 9.8	BOO: 67.8 ± 21.5; BOO + DU: 51.6 ± 8.0
	Matsukawa et al. 2021 [16]	BCI ≤ 100 and BOOI > 40	BOO: BCI > 100 and BOOI > 40, DU: BCI ≤ 100 and BOOI ≤ 40							DU + BOO + : 84.9 ± 9.1	BOO: 121 ± 22; DU: 68.8 ± 18.3	DU + BOO + : 51.6 ± 8.0	BOO: 67.8 ± 21.5, DU: 27.1 ± 9.8
6	Takahashi et al. 2021 [32]	BCI < 100 and BOOI ≤ 40	BOO: BOOI > 40 and BCI ≥ 100										
7	Namitome et al. 2020 [33]	BCI < 100	BOO: BOOI > 40										
8	Matsukawa et al. 2023 [34]	BCI < 100 and BOOI < 40	BOO: (BCI ≥ 100, BOOI ≥ 40), DU + BOO: (BCI < 100, BOOI ≥ 40), and non-DU + non-BOO: (BCI ≥ 100, BOOI < 40)							68.6 ± 19.2	BOO: 128.4 ± 20.1; DU + BOO: 86.4 ± 11.0, non-DU nonBOO: 120.0 ± 16.4	26.0 ± 9.3	BOO: 70.8 ± 23.9, DU + BOO + : 53.7 ± 9.4, non-BOOnonDU: 26.4 ± 13.5
9	Kim et al. 2019 [35]	BCI < 100	BOOI > 40							60.4 ± 30.7	121.9 ± 32.4	13 ± 13.4	63.7 ± 34.5
10	Ishikawa et al. 2023 [17]	BCI < 100 and BOOI < 40	DO: uninhibited detrusor contraction with an increasing amplitude of ≥ 10 cm H20 during the filling phase			median 7 (4–8)	median 7 (6–11)			median 71 (60–78.5)	median 113 (99–126.5)	median 27 (24.5–32)	median 51 (40–72.5)
11	Krishnan et al. 2021 [18]	BCI < 100, no DO on UDS	age and sex-matched healthy persons (no UDS)	32.3 ± 21.9		4.0 ± 3.2				57.9 ± 27.6			
12	Luo et al. 2017 [36]	Pdet.max < 50 cmH2O						33.45 ± 14.15	97.81 ± 37.3				
13	Lee et al. 2022 [13]	BCI < 100, BVE < 90% and BOOI < 40	BOO: BOOI > 40	35.8 ± 6.7	67.3 ± 26.2	8.3 ± 4.1	9.1 ± 4.3			69.1 ± 21.0	100.1 ± 29.7	22.7 ± 6.3	52.8 ± 28.3
14	Bang et al. 2022 [15]	BCI < 100	BOO: BOOI > 40			11 .09	14.67			78.38	127.66	26.22	33.01
15	Lee et al. 2016 [12]	BCI < 100 and BOOI < 20	BOO: BOOI ≥ 40	13.6 ± 0.7	34.7 ± 1.2							15.3 ± 16.5	56.4 ± 16.8
16	Yoldas 2022 [37]	BCI < 100 and BOOI > 40	BCI > 100 and BOOI > 40	34.1 ± 21.31	101.1 ± 40.02	4.2 ± 3.96	6.5 ± 3.98			48.8 ± 27.21	132.5 ± 37.83	20.0 ± 8.82	88.0 ± 40.69
17	Wu et al. 2024 [38]	PIP < 100		32.7 ± 7.8	49.3 ± 11.6	4.4 ± 1.2	13.0 ± 3.0						
18	Garbas et al. 2024 [39]	BCI < 100	BOO: BOOI > 40	median 37 (20–51)	median 80 (70–93)	median 6.6 (4.5–8.8)	median 8.1 (6.6–11.1)	median 46.5 (29–58.5)	median 101 (84–123)	median 78.5 (57.8–88)	median 124.5 (112.5–141.5)	median 22 (7.5–40.6)	median 64.6 (52.6–80.8)

*BCI* bladder contractility index, *BOO* bladder outlet obstruction, *BOOI* bladder outlet obstruction index, *DO* detrusor overactivity, *DU* detrusor underactivity, *PFS* pressure flow study, *PIP* projected isovolumetric pressure, *PVR* post-void residual, *UDS* urodynamic study, *BVE* bladder voiding efficiency

Three studies employed diagnostic criteria for DU that differed from the commonly used BCI and/or BOOI. One study defined DU by exclusion, identifying DU patients with PVR > 30 ml but without BOO or dysfunctional voiding in PFS [31]. Another study defined DU as Pdet.max < 50 cmH2O on PFS [36]. A third study used a combination of criteria: BCI < 100, BOOI < 40, and bladder voiding efficiency (BVE) < 90% [13]. Medication potentially affecting detrusor function was specified in 14 studies. Studies included patients without LUTS therapy (*n* = 8), LUTS therapy was stopped 8 weeks prior to UDS (*n* = 1) or 3 days prior to UDS (*n* = 1) or patients were on constant medication (*n* = 4). One study included patients with LUTS refractory to medication, without detailing their current medication intake, and one study included healthy controls without LUTS or medication. Comorbidities were reported in only 6 studies, with the most prevalent being hypertension (HTN) and diabetes mellitus (DM) (see Table 2 for details).

### The investigated non-invasive diagnostic methods

Diagnostic methods, their descriptions, sensitivity, and specificity were demonstrated in Table 4.

**Table 4:** Characteristics of the non-invasive diagnostic methods

No	Study	Diagnostic method	Measurement method	Threshold/cut-off value	Sensitivity	Specificity	PPV	NPV	Accuracy (other)	Subgroup analysis	Feasibility of the method	Limitations of the study
1	Kiba et al. 2022 [30]	Smaller PV		PV: 39 ml =	75%	67%	50.2%	85.8%		no		1. Retrospective study. 2.Severe cases included. 3. The BCI and BOOI criteria used for DU diagnosis. 4. 13% of patients had DM
2	De Nunzio et al. 2020 [14]	visual nomogram including: Age, PV, BWT and Qmax on UFL	BWT was measured on the bladder filled to 150 mL, with suprapubic US using the 3.5 MHz convex probe, in the horizontal direction at maximum magnification. PV was measured with transrectal US using the ellipsoid formula (p/6 width height depth of prostate). Analysis of UFL (Qmax) and clinical data (age)	ex. score of 14 corresponds to 70% probability of DU					AUC = 0.82; net benefit in the range of probabilities between 10 and 80%;	severe LUTS group: AUC = 0.84	1. Useful for identifying patients at high risk of DU who should undergo PFS. 2. Patients at low risk of DU could be spared of an invasive test. 3. Could be used in neurological patients (after an external validation)	1. A retrospective study (data collected prospectively) 2. Applicable to males with LUTS, a small prostate nonreceiving medical treatments 3. A small percentage of patients with severe LUTS (30%)
3	Matsukawa et al. 2020 [11]	presence of "sawtooth and interrupted" waveform on UFL	When the flow curve showed an interrupted shape with ≥ 3 discrete peaks, as well as with a repeated decrease of urine flow to zero followed by an increase, during micturition	yes vs no	80%	87.2%	76.8%	89.2%		no	Useful, high-integrity factor to predict DU	1. A retrospective study 2. Difficulty evaluating flow pattern 3. DU defined as BCI < 100 and BOOI < 40, however, the prevalence of DU was reported to be different according to other diagnostic criteria based on a urodynamic measure
		lower IPP	IPP = the vertical distance from the tip of the protrusion to the circumference of the bladder at the base of the prostate gland using transabdominal US longitudinal imaging with a bladder urine volume of approximately 150–200 mL	8.2 mm	77%	73%	60.2%	85.8%		no		
		lower BVE		70%	66%	57%	45.1%	76.1%		no		
4	Rademakers et al. (FORCE Research Group) 2017 [31]	DWT on US in combination with BC	US DWT measurements were taken at the anterior bladder wall with a 7.5 MHz US array and bladder filling ≥ 250 ml. A mean of three DWT measurements was used for the analysis. BC = [VV + PVR] on UFL	DWT ≤ 1.23 mm with BC > 445 ml	42%	100%	100%	85%	accuracy of 87%, a likelihood ratio of a positive test result (DU) of 42, and a likelihood ratio of a negative test result (no-DU) of 0.58	no	Patients with DU could be identified with the two non-invasive parameters and without urodynamic investigation	1. A retrospective study. 2. Small group of patients. 3. Only applicable for male adult male patients (no females, no children with PVR)
5	Matsukawa et al. 2021 [16]	AI based model (for DU diagnosis)	Model established using tenfold stratified crossvalidation and data augmentation		79.7%	88.7%	75.9%	90.7%	accuracy of 84%	no	The AI system could diagnose LUTD by calculating BCI and BOOI directly from the UFL waveform and could diagnose DU + BOO	1. The AI system did not learn normal patient waveforms. 2 Insufficient evaluation of reproducibility in the same person. 3. Women not analyzed
		AI based model (for DU + BOO + diagnosis)			50.8%	83.2%	49.7%	83.8%	diagnostic predictive value = 50.8%	no		
6	Takahashi et al. 2021 [32]	3-parameters nomogram composed of: 1) IPSS Q4, 2) PV 3) age; 1 point for each factor	PV measured by transrectal or abdominal US; IPSS questionnaire and clinical data analysis (age)	≥ 74 years old, PV ≤ 34.8, and IPSS Q4 ≤ 1					probability of DU in patients with 3 significant factors- 77%, with 2 factors- 67%, with 1 factor- 54%, with 0 factors- 24%	no	Selected three factors could be useful for differentiating DU from BOO	1. A retrospective study 2. Excluded patients with urinary retention and those who could not void > 50 ml in a PFS 3. PV was evaluated using transrectal or abdominal ultrasonography (variability) 5. Even if all three factors are present, 34% of patients still have BOO and 23% of patients still do not have DU. 6. Among non‐DU patients with all three factors, 76% of the patients had a BCI ≤ 120 (borderline DU)
7	Namitome et al. 2020 [33]	5-parameter scoring model, including: 1) age, 2) PV, 3) IPSSQ4, 4) IPSSQ5, 5) Qmax on UFL;	PV was measured by transrectal or abdominal US; IPSS questionnaire, UFL analysis, clinical data analysis (age); 1) Age 50–74- 0points, > = 75—2 points; 2) PV: < 30 ml- 5 points, > = 30 and < 50- 2 points, > = 50 ml 0 points; 3) I-PSS Q4 < = 2—1 point, 4) I-PSS Q5 > 2- 1 point; 5) Qmax on UFL- < = 5- 2points, > 5 and < = 10 0 1 point, > 10- 0 points						c-index: 0.724; points and predicted probability of DU: 0 points- 12%; 11 points- 88%,	no	The study included patients with BOO and DO, which is important as DU, BOO and DO often overlap clinically	1. No external validation. 2. The effect of medications not considered. 3. Excluded patients with urinary retention or those who could not void > 50 ml in the PFS
8	Matsukawa et al. 2023 [34]	AI-developed UFL-based parameter: 1) lower first peak flow rate	The first peak flow: the point in the voiding wave shape at which the subsequent urine flow rate decreases. Even if the gradient of the urine waveform decreased, it was not judged as the first peak flow unless the gradient was negative	6.5 mL/s	72%	86%	64.7%	89.6%	AUC = 0.82	no	The first peak flow can be easily assessed in clinical practice	1. Study required 0.1-s interval measurements of the UFL waveforms. 2. A retrospective study. 3. DU was defined as BCI < 100 and BOOI < 40. 4. The first peak flow parameters may not be useful, when VV is too low (< 50 mL). 5. Difficult to diagnose the severity of DU or exactly distinguish DU from BOO + DU
		AI-developed UFL-based parameter: 2) lower ratio of the first peak flow rate to Qmax	Calculated as the first peak flow rate/Qmax	0.8	76%	83%	61.6%	90.6%	AUC = 0.85	no		
9	Kim et al. 20198 [35]	Higher score in DUA-SQ: 8-questions questionnaire based on symptoms	8 parameters to assess; each scored 0,5,10 or 15 points	45 points	95.8%	95.4%	95.8%	95.4%	AUC = 0.99	no		1. A retrospective study 2.Difficult to detect patients who overlap with DU and BOO (designed for the differential diagnosis)
10	Ishikawa et al. 2023 [17]	Lower total serum adiponectin level	Total serum adiponectin levels were measured using a particle-enhanced turbidimetric immunoassay at a commercial laboratory using a latex bead-immobilized anti-adiponectin polyclonal antibody	7.9 μg/mL	79%	90%	79.6%	89.7%	AUC = 0.85	no	Adiponectin can be easily measured with good reproducibility	1. Other factors associated with lifestyle-related diseases and MetS not evaluated. 2. A control group of healthy participants or a patients with different BMI and lifestyle-related diseases not included 3.Patients taking anticholinergic drugs, statins and anti-inflammatory drugs not excluded 4. DU defined as a BCI < 100 and a BOOI < 40 and most patients had 20 < BOOI < 40. 5. Some patients in the non-DU group with BOOI > 40 but BCI < 100 6. A cross-sectional study
11	Krishnan et al. 2021 [18]	Higher NO/ATP ratio in urine samples	ELISA kits were used to estimate NO and ATP levels from urine	2.06	88.5%	88.9%	92.0%	84.2%	accuracy of 88.6%, AUC = 0.91	no	Spot urinary levels of NO and ATP might help reach a diagnosis of DU for indeterminate urodynamic findings or help completely avoid UDS	1. Small sample size 2. DU of different etiologies 3. Control subjects labeled as usual based on history- the UDS was not performed 4. DU defined with BCI < 100
12	Luo et al. 2017 [36]	Smaller PV combined with higher PVR	PV and PVR were measured using abdominal US. Prolate ellipsoid formula (volume = π/6 × length × width × height) was used to obtain the PV	PV = 46.05 ml and PVR = 147 ml	77.8%	73.7%	35.8%	94.6%	AUC = 0.77	no		1. Not all clinical determinants of DU assessed. 2. Only Pdet. max used as the criterion for DU. 3. The threshold of Qmax might be inaccurate as a result of the low values. 4. Small sample size 5. Results based on Asian men
13	Lee et al. 2022 [13]	Smaller ratio of DMT and BWT = (DMT/BWT × 100) %	US measurements of the DMT and BWT were performed every 50 mL during filling in a cystometric study up to 500 mL or MCC. DMT and BWT were measured at the anterior wall of the bladder with the patient in the supine position	< 47.5% of the thickness ratio at 20% of the MCC	86.7%	64.3%	45.4%	93.4%	AUC = 0.76	no	The thickness ratio remained relatively constant up to 50% of the MCC; thus, measuring the thickness ratio < 50% of the MCC would be more accurate and convenient	1. Small number of DU patients 2. The age of the two control groups not matched 3. Difficult to repeat bladder filling for patients diagnosed with DU due to UDS invasiveness 4. Operator-dependent 5. Measuring DMT and BWT by US is challenging below 50% of the MCC
14	Bang et al. 2022 [15]	Deep learning-based diagnostic platform based on UFL graphs analysis	UFL graph was extracted automatically using the ABBYY Flexicapture® image capture program. A convolutional neural network (CNN) was applied. A fivefold cross-validation average value of the AUROC curve was chosen as an evaluation metric. The corresponding average precision-recall (PR) curves were provided. The average AUROC scores of DUA were measured using fivefold cross-validation and the best score was obtained with fine-tuned VGG16 network		65.9%	68.9%	67.9%	67.0%	AUROC score = 73.30% (mAP = 0.70)	no		1. Low prediction rate (over 70%). 2. The capacity to set the basis for model predictions confined due to the absence of external data. 3. Excluded patients with both BOO and DU
15	Lee et al. 2016 [12]	Smaller DeltaQ = (Qmax—Qmean) on UFL	UFL analysis	6.65 mL/s	71.3%	70.3%	67.5%	74.0%	AUC = 0.81	no		1. A retrospective study. 2. Only patients with DU or BOO, men with mixed urodynamic problems were excluded. 3. Possibly other conditions affect DeltaQ. 5. UDS interpreted by a single urologist. 6. A single institution study
		Smaller Qmax on UFL	UFL analysis	11.05 mL/s	69%	68.5%	65.3%	71.9%	AUC = 0.76			
16	Yoldas 2022 [37]	Higher BVE on UFL (for men > 80 years old)*	BVE = VV on UFL/pre-void BC measured on US	46%	93%	60%	67.6%	90.5%	AUC of max. diagnostic value for VE = 0.771	yes; age > 80 years old	Applicable for men over 80 years old	1. The BOOI 20–40 not examined. 2. A cut-off of 40 for AG-number used
17	Wu et al. 2024 [38]	Older age		66.5 years	72%	46%	54.6%	64.5%	AUC = 0.71	no		1. Limited number of participants. 2. Incomplete incorporation of inflammation markers. 3. The hematological markers oversimplified and did not provide accurate representation of patient’s condition
		Lower PSA level		2.90 ng/mL	30%	49%	34.7%	43.7%	AUC = 0.37			
18	Garbas et al. 2024 [39]	10-factor model, including nocturia, intermittency, weak stream, straining points in CLSS, slow stream points in CLSS, incomplete emptying points in CLSS, PVR ratio, and fluctuating, fuctuating-intermittent and intermittent UFL curve shapes	medical history, CLSS questionnaire, interpretation of UFL, including curve shape analysis		75.8%	62.4%	71.9%	67.0%	C-index: 0.783	no	Can be useful during the initial ambulatory visit	1. A retrospective study 2. Excluded patients who could not void > 150 ml on UFL, including those with urinary retention 3. A single geographic and ethnic population study, performed on white Polish males 5. PV not evaluated 6. Some patients on medication affecting voiding

*AI* artificial intelligence, *AUC* area under the curve, *AUROC* area under the receiver operating characteristic curve, *BC* bladder capacity, *BCI* bladder contractility index, *BMI* body mass index, *BOO* bladder outlet obstruction, *BOOI* bladder outlet obstruction index, *BE* bladder voiding efficiency, *BWT* bladder wall thickness, *CLSS* core lower urinary tract symptom score, *DM* diabetes mellitus, *DWT* detrusor wall thickness, *DO* detrusor overactivity, *DU* detrusor underactivity, *DUASQ* detrusor underactivity symptom questionnaire, *IPP* intravesical prostatic protrusion, *IPSS* International Prostate Symptom Score, *LUTD* lower urinary tract dysfunction, *LUTS* lower urinary tract symptoms, *MCC* maximal cystometric capacity, *PFS* pressure flow study, *PSA* prostate specific antigen, *PSAD* prostate-specific antigen density, *PV* prostate volume, *PVR* post-void residua, *UDS* urodynamic study, *UFL* uroflowmetry, *US* ultrasound, *VV* voided volume

#### Nomograms and scoring models

Five studies developed and applied nomograms or scoring models for DU diagnosis, comparing patients with DU to non-DU individuals. One study introduced a visual nomogram combining age, prostate volume (PV), bladder wall thickness (BWT) measured via ultrasound, and maximum flow rate (Qmax) on UFL. This model demonstrated a 70% predictive probability of DU for patients scoring 14 points, which increased to approximately 93% when the total score reached 16 points [14]. Another study used a three-parameter nomogram composed of International Prostate Symptom Score (IPSS) question 4 (Q4), PV and age. A patient aged 74 or older, with a PV of 34.8 ml or less and an IPSS Q4 score of 1 or lower, scored 3 points in the nomogram, which corresponded to a 77% probability of DU [32]. Namitome et al. developed a five-parameter scoring model that incorporated age, PV, IPSS Q4 and Q5, and Qmax on UFL. In this model, scores of 1, 2, or 5 were assigned to each variable. For example, a 75-year-old male patient (2 points) with an IPSS Q4 score of 2 (1 point), an IPSS Q5 score of 3 (1 point), a PV of 30 ml (2 points) and a Qmax on UFL of 5 ml/sec (2 points) would have a total score of 8 points, corresponding to 72% probability of DU [33]. One study employed a nomogram based on PV and PVR, achieving 77% sensitivity and 73% specificity for DU diagnosis [36]. Another study developed a 10-factor model that incorporated symptoms, the Core Lower Urinary Tract Symptoms score (CLSS), PVR ratio (calculated as the percentage of PVR to bladder volume [voided volume + PVR]), and UFL curve shapes. The ten factors included nocturia, intermittency, weak stream, straining points in CLSS, slow stream points in CLSS, incomplete emptying points in CLSS, PVR ratio, and fluctuating, fluctuating-intermittent and intermittent UFL curve shapes. This model demonstrated sensitivity of 75.8%, similar to the abovementioned models, but had a lower specificity of 62.4% [39].

#### UFL analysis

Four studies utilized various UFL parameters as a diagnostic method for DU. One study investigated the presence of "sawtooth" and "interrupted" waveforms on UFL, characterized by an interrupted flow curve with three or more discrete peaks, as well as repeated decrease in urine flow to zero followed by an increase, during micturition. This method achieved a sensitivity of 80% and a specificity of 87.2%, for differentiating DU from BOO patients. Additionally, the study assessed bladder voiding efficiency (BVE), defined as the ratio between voided volume and total bladder capacity [9]. However, it performed worse, with a sensitivity of 66% and specificity of 57% [11]. Another study also evaluated the utility of BVE, showing that DU could be diagnosed with a 93% sensitivity and a 60% specificity in men over 80 years old, with UFL measurements indicating voiding efficiency higher than 46% [37]. Lee et al. tested a DeltaQ parameter, calculated as Qmax – Qmean on UFL. The area under the curve (AUC) for DeltaQ (0.806) was significantly higher than that from Qmax (0.763) and Qmean (0.574). With a cutoff value of 6.65 mL/s, it had a sensitivity of 71.3% and specificity of 70.3% [12]. Qmax was also evaluated in another study, showing a sensitivity of over 80% and specificity of 63%. However, it performed worse than the combined PV and PVR for predicting DU [36].

#### Ultrasound parameters

Three studies focused on ultrasound parameters. Two investigated detrusor wall thickness (DWT), bladder wall thickness (BWT), or their derivatives, and one assessed intravesical prostatic protrusion (IPP). The FORCE Research Group evaluated DWT in combination with bladder capacity (BC) as a method for diagnosing DU. DWT measurements were taken at the anterior bladder wall, using a 7.5 MHz ultrasound array with the bladder filled to over 250 mL. A mean of three DWT measurements was used for the analysis. BC was calculated as BC = [voided volume + PVR)] on UFL. A DWT of 1.23 mm or less, combined with BC of over 445 mL demonstrated a 100% specificity for DU diagnosis but had poor sensitivity of only 42% [31]. In contrast, Lee et al. studied the ratio of detrusor muscle thickness (DMT) to BWT, calculated as (DMT/BWT × 100) %, which showed better sensitivity but lower specificity. A thickness ratio of less than 47.5%, measured at 20% of the maximal cystometric capacity (MCC), provided a sensitivity of over 86% and a specificity of approximately 64%. In this study, ultrasound measurements of the DMT and BWT were performed every 50 mL during filling in a cystometric study up to 500 mL or MCC. Both DMT and BWT were measured at the anterior wall of the bladder with the patient in the supine position. BWT was defined as the width of the bladder wall, including the two thin hyperechoic layers of the mucosa, while DMT was specified as the width of the hypoechoic space, excluding the hyperechoic layers [13]. The third study investigated IPP, which was calculated as the vertical distance from the tip of the protrusion to the circumference of the bladder at the base of the prostate gland, using transabdominal ultrasonographic longitudinal imaging, with a bladder urine volume of approximately 150–200 mL. In this study, IPP had a significant correlation with BCI and was a useful predictor of DU, with a sensitivity of 77% and a specificity of 66% [11].

#### Biomarkers

Three studies investigated potential biochemical markers for DU diagnosis, focusing on those detectable in blood and urine samples. Ishikawa et al. examined total serum adiponectin levels, measured using a particle-enhanced turbidimetric immunoassay, as a potential predictor of DU. This method demonstrated a sensitivity of 79% and a specificity of 90% [17]. Krishnan assessed the NO/ATP ratio in urine samples with the use of ELISA kits, comparing DU patients to healthy controls. This marker reached both sensitivity and specificity levels exceeding 88% for DU diagnosis [18]. In contrast, the third study showed that several hematological serum markers, including Prognosis Nutrition Index (PNI), Systemic Immune Inflammation Index (SII), Neutrophil to Lymphocyte Ratio (NLR), and Platelet to Lymphocyte Ratio (PLR), were not significant predictors of DU [38].

#### Artificial intelligence and machine learning

Three studies incorporated AI and deep learning to diagnose DU. Matsukawa et al. developed an AI-based model established using only UFL data, tenfold stratified cross-validation, and data augmentation, which demonstrated a sensitivity of almost 80% and a specificity of over 88% for DU diagnosis and over 50% sensitivity and 83% specificity for DU + BOO + diagnosis [16]. Another study investigated parameters developed with the help of AI based on UFL, including first peak flow rate and the ratio of the first peak flow rate to Qmax. The first peak flow was defined as the point in the voiding wave shape at which the urine flow rate decreases. Even if the gradient of the urine waveform decreased, it was not judged as the first peak flow unless the gradient was negative. Both parameters showed good diagnostic performance, with sensitivities of 72% and 76%, and specificities of 86% and 83%, respectively [34]. The third study utilized a deep learning diagnostic platform based on UFL graphs analysis, achieving a sensitivity of over 65% and specificity of 68% [15].

#### Symptom questionnaires

One study developed a new questionnaire, the DUA Symptom Questionnaire (DUA-SQ), consisting of eight targeted questions addressing urinary symptoms. These questions included erectile dysfunction, long duration of void, nocturia, abdomen distention, sensation of void, straining, bowel dysfunction, and weak stream. Each symptom was assessed based on its severity and was assigned a score of 0, 5, 10, or 15 points. The questionnaire demonstrated a sensitivity of 95.8% and a specificity of 95.4% for predicting DU in patients who scored more than 45 points [35].

#### Additional parameters

Other studies evaluated various parameters, including PV, age, and PSA level. Kiba et al. found that smaller PV, older age, and less urgency may be clinical features of DU. The study identified 39 mL as the optimal cutoff value for PV for the diagnosis of pure DU, with a sensitivity of 75% and a specificity of 67% [30]. Wu et al. examined a PSA cut-off value of 2.9 ng/mL; however, the sensitivity and specificity were low, reaching 30% and 49%, respectively. Age was also considered an ancillary factor in assessing the likelihood of DU [38].

### Risk of bias assessment

**Fig. 2:**
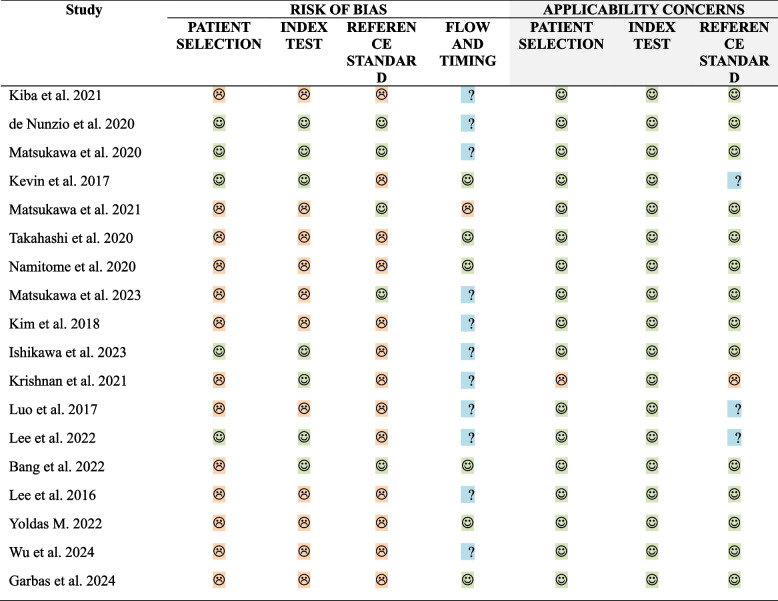
Risk of bias and applicability concerns assessment of the studies included in the systematic review using QUADAS-2 tool

**Fig. 3:**
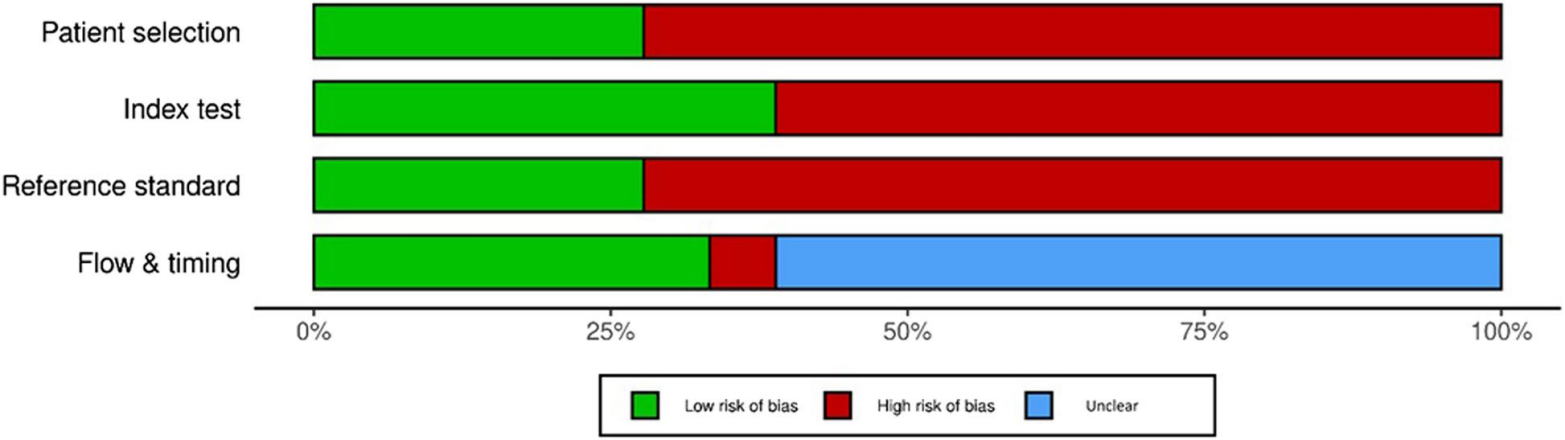
A summary plot of the risk of bias for main domains of the studies created with the use of the Robvis tool [29]

When assessing the risk of bias using the QUADAS-2 tool, the risk of bias in the patient selection domain was evaluated as “low” for prospectively recruited, retrospectively analyzed cross-sectional studies [11, 13, 14, 17, 31], and “high” for retrospective cross-sectional and case–control studies. In one case–control study [18], applicability concerns were rated as”high” for patient selection and the reference standard, as the control group consisted of healthy individuals without LUTS who did not undergo PFS. Overall, due to the poor quality of the studies and data heterogeneity, the meta-analysis was not performed, and only a qualitative synthesis was conducted. For a detailed risk of bias assessment, see Figs. 2 and 3.

## Discussion

In this systematic review, we conducted a comprehensive analysis of non-invasive tests for diagnosing DU in men with non-neurogenic LUTS. Our review underscores the diversity of emerging non-invasive diagnostic methods, which include ultrasound parameters, biomarkers, uroflowmetry results, symptom questionnaires, and clinical characteristics, as well as models developed with the help of AI and machine learning. Notably, the methods identified as having the highest sensitivity and specificity were the symptom questionnaire DUA-SQ and DWT measured with ultrasound in combination with BC, respectively. Furthermore, our review highlights the potential role of machine learning and AI in developing predictive models for DU. Our study also reveals the problem of significant variability in the diagnostic criteria for DU. This underscores the need for developing standardized diagnostic criteria in order to improve both the consistency and reliability of DU diagnoses. To our knowledge, this is the first systematic review that comprehensively summarizes the up-to-date data on the non-invasive tests for DU diagnosis in men with non-neurogenic LUTS, indicating their accuracy, strengths, and limitations.

UDS, currently considered the gold-standard for diagnosing DU, poses several inconveniences for patients, including embarrassment, pain, and dysuria. UDS is also not devoid of complications, such as urinary retention, macroscopic hematuria, or urinary tract infection [40], which can be dangerous for immunocompromised patients. On the contrary, non-invasive diagnostic methods, especially UFL analysis [11, 12], symptom questionnaires [35] and diagnostic nomograms [32, 33, 36, 39], offer a more convenient, non-traumatic, and safer alternative. These methods rely on easily accessible parameters (e.g., UFL, IPSS, age, PV), facilitating quicker diagnoses, even in uncooperative patients and during initial outpatient visits. Additionally, symptoms questionnaires and diagnostic nomograms are intuitive, easy to follow and integrate into daily clinical practice, unlike UDS, which requires highly trained experts for accurate interpretation and diagnosis.

The integration of AI into diagnostic methods has only recently begun but holds significant promise for the future. Matsukawa et al. were pioneers in developing an AI system capable of diagnosing lower urinary tract dysfunction (LUTD) by calculating BCI and BOOI directly from UFL waveforms. Preliminary findings indicated that the AI system diagnosed DU or BOO with high sensitivity and specificity, utilizing only limited data. When the diagnostic accuracy of the AI system was compared to that of three urologists, the AI system outperformed the clinicians, achieving a sensitivity of 80% and a specificity of 100% for DU diagnosis, whereas the urologists had a sensitivity of 46.7% and a specificity of 80% [16]. AI also contributes to the development of new diagnostic parameters derived from established tools like UFL. In another study, Matsukawa et al. proposed using the first peak flow rate and the ratio of the first peak flow rate to Qmax in UFL as diagnostic markers for DU. Both parameters were developed with AI assistance and can be easily implemented into clinical practice [34]. Additionally, deep learning diagnostic platforms, such as one developed by Bang et al., can interpret UFL results and assist clinicians in decision-making by quickly analyzing and comparing vast data sets [15].

The ongoing search for serum or urine biomarkers for DU is also promising, as they could provide more objective diagnostic tools with straightforward interpretation. Ishikawa et al. studied adiponectin, a beneficial adipokine with reported anti-inflammatory and anti-atherogenic properties [41, 42], as a potential DU predictor. Adiponectin was easy to measure and had good reproducibility, with a cutoff value of 8 μg/mL, providing high sensitivity and specificity for DU diagnosis. These findings suggest that adiponectin could serve as a useful screening marker for bladder dysfunction and may potentially predict treatment efficacy for LUTS, including surgical interventions. Nevertheless, the precise relationship between urodynamic parameters and adiponectin remains unclear and warrants further investigation [17]. Another potential biomarker is the NO/ATP ratio in urine samples. Since ATP and nitric oxide (NO) released from the urothelium have been shown to affect the afferent stimuli arising from the bladder [43], Krishnan et al. investigated the NO/ATP ratio in patients with DU, comparing it to healthy, age- and sex-matched volunteers without LUTS. Although the study found no correlation between urinary ATP and NO levels, and urodynamic parameters, the NO/ATP ratio showed excellent discrimination between DU patients and controls, with an area under the curve (AUC) of 0.91. This indicates that urinary NO and ATP levels warrant further exploration as potential DU biomarkers. If implemented, this method would be entirely non-invasive, requiring only urine samples from patients [18].

Despite the strengths of the studies included in this review, several limitations and concerns about the methods, their quality, and reliability remain. Most studies were retrospective, single-center, and conducted in Asia, which introduces selection bias. Moreover, none of the studies published after 2023 applied the newly introduced ICS-SUFU standards on PFS analysis [10], likely because their research began before the updated guidelines took effect. Future studies that avoid relying on a strict DCI < 100 threshold are needed to more precisely assess the detrusor function. Given the limited utility of a single cutoff at 100 for defining a weak detrusor, using DCI as a continuous variable or introducing an additional “very weak” threshold (DCI < 50) may provide greater clinical relevance and utility in daily practice. Additionally, none of the diagnostic methods have undergone external validation, further limiting their applicability. Although Krishnan et al. conducted a case–control study, the control group consisted of healthy, age- and sex-matched volunteers without any LUTS (indicated by a zero IPSS score), who did not undergo UDS. This may have led to overly optimistic findings [18]. Several studies did not account for patient medications [15, 32, 34, 35], included patients on drugs affecting detrusor function [13, 30, 33, 39] or only ceased medication three days prior to UDS [37]. Overlooking the impact of medications on bladder function might have affected the studies’ results. Additionally, some researchers excluded patients who could not void over 150 mL during UFL [15, 37, 39] or whose total voided and residual urine volumes in UFL were under 100 mL [34]. These exclusions limit applicability, as patients with the most severe LUTS were omitted from the study cohort. It is important to note that some diagnostic tools were restricted to specific patient subgroups. For example, Yoldas developed a diagnostic tool solely for men over 80 years old [37], while de Nunzio et al. created a method applicable to males with LUTS, a small prostate (median 30 g) not receiving medical treatment for LUTS [14]. This suggests that a universal tool applicable to all patients may not exist. Instead, various non-invasive diagnostic methods tailored for specific subgroups might be necessary.

Moreover, even though some methods seem quick and easy to follow, such as the newly developed DUA-SQ, proposed by Kim et al. [35], they are highly subjective. Its results depend on a patient’s understanding of symptoms, current well-being, and patient-clinician dialogue, which can lead to significant variability.

Some methods rely on ultrasound to measure BWT [14, 31] or DMT/BWT ratio [13]. To standardize measurement conditions, BWT was assessed in a bladder prefilled with a calculated volume of fluid via catheterization. This approach is suboptimal, as each catheterization increases the risk of urinary tract infection, especially in patients with neutropenia or renal diseases [44]. Moreover, ultrasound diagnoses are operator-dependent, and require consistent training to ensure reproducibility of results.

Other methods are based on UFL analysis, which has already demonstrated diagnostic utility in various urological conditions. Despite the quick and easy implementation of UFL, recent data suggest that several factors, including diurnal, postural, locational, and operator variability, may introduce variability in test results, affecting its overall diagnostic value [45]. Matsukawa et al. faced challenges in precisely evaluating UFL flow patterns, particularly "sawtooth" and "interrupted" waveforms, noting that interpretations could vary between clinicians [11]. A study by Lee et al. identified DeltaQ as the most specific and sensitive UFL parameter, however, it exclusively included patients with DU or BOO, excluding men with mixed urodynamic issues. Limited data on other conditions that may also affect DeltaQ highlights the need for further prospective trials [12].

Regarding DU biomarkers, Ishikawa et al. introduced adiponectin as a possible DU predictor. However, this study did not evaluate other major pathological factors associated with lifestyle-related diseases and metabolic syndrome, such as leptin, TNF-α, or interleukin-6. Furthermore, the study did not include patients with lifestyle-related diseases or varying BMI—the median BMI of 23.0 appeared low compared to Western populations. Lastly, it remains unclear whether serum adiponectin levels increase following LUTS treatment or whether elevated adiponectin levels improve bladder function [17]. Wu et al. aimed to analyze the potential of systemic inflammatory markers in predicting DU in BPH patients with LUTS and low PV [38]. The study posited that inflammatory markers in the bloodstream might correlate with chronic bladder ischemia, contributing to DU. However, their role as potential markers was not confirmed. It is essential to recognize that identifying new biomarkers and incorporating them into clinical practice can be costly and time-consuming. Additionally, the pathogenesis of DU is multifactorial, involving a complex interplay of aging, neurogenic, myogenic, and iatrogenic factors [46], making it unlikely to find a single universal biomarker for DU.

Recent advancements in AI have also impacted DU diagnostic methods. The initial study by Matsukawa et al. in 2021, which developed an AI-based model, encountered several limitations. The AI system did not learn normal patient waveforms, and the evaluation of reproducibility within the same patient was insufficient. Diagnosing coexisting pathological conditions, such as concurrent DU and BOO, also proved challenging [16]. In 2023, Matsukawa et al. utilized AI to develop new specific parameters derived from available UFL data to aid in DU diagnosis. However, this study also had limitations, including an analysis restricted to only 266 cases due to the requirement for precise 0.1-s interval measurements of urinary flow waveforms. Additionally, when the voided volume is too low, the UFL waveform often appears monophasic, regardless of LUTS pathophysiology, which may render parameters related to the first peak flow ineffective for differentiating DU [34]. Bang et al., on the other hand, used deep learning in order to create a diagnostic platform based on UFL graphs analysis. However, the prediction rate was only slightly over 70%, and the capacity to set the basis for model predictions confined due to the absence of external data [15].

Future research on diagnostic methods should not only focus on identifying non-invasive markers for diagnosing DU but also on evaluating the degree of detrusor weakness by considering the DCI as a continuous variable. A key question is whether these methods can determine a patient’s candidacy for surgical intervention and accurately predict likely benefit. In the recent UPSTREAM trial, patients with LUTS who had BCI greater than 123.0 showed more favorable outcomes following surgery [47], underscoring the need to develop non-invasive tools that can reliably identify those most likely to benefit from surgical treatment.

Several non-invasive methods included in this analysis, particularly those that are straightforward to use in clinical practice and have high sensitivity – such as the DUA-SQ questionnaire [35] or the "sawtooth and interrupted" waveform on UFL [11] – could serve to preselect patients for invasive UDS, after external validation of these methods. This approach would improve the diagnostic process and reduce costs. Moreover, combining multiple existing techniques into a single diagnostic algorithm and evaluating its overall accuracy could further enhance clinical decision-making for DU.

## Conclusions

Non-invasive diagnostic methods, including ultrasound parameters, biomarkers, UFL analysis, symptom questionnaires, clinical characteristics, and AI integration, show promise in diagnosing DU and offer safer, more convenient alternatives to invasive UDS. However, many methods face limitations such as a lack of accuracy and external validation, as well as variability in test conditions, which affect their reliability and applicability. Our work provides a valuable resource for clinicians seeking to optimize diagnostic strategies for DU by consolidating evidence on the efficacy and limitations of these non-invasive tests. Furthermore, our analysis highlights important areas that require further investigation to refine existing methods, examine their applicability, and create diagnostic tools customized for specific patient subgroups.

## Data Availability

The datasets used and/or analysed during the current study are available from the corresponding author on reasonable request.

## Abbreviations

DU: Detrusor underactivity
LUTS: Lower urinary tract symptoms
ICS: International Continence Society
UAB: Underactive bladder
OAB: Overactive bladder
BOO: Bladder outlet obstruction
UDS: Urodynamic study
PFS: Pressure flow study
BCI: Bladder Contractility Index
BOOI: Bladder outlet obstruction index
UFL: Uroflowmetry
BWT: Bladder wall thickness
DMT: Detrusor muscle thickness
AI: Artificial intelligence
PRISMA: Preferred Reporting Items for Systematic Reviews and Meta-analyses
PICO: Population, Intervention, Control, Outcome
PPV: Positive predictive value
NPV: Negative predictive value
QUADAS-2: Quality Assessment of Diagnostic Accuracy Studies 2
BE: Bladder voiding efficiency
HTN: Hypertension
DM: Diabetes mellitus
PV: Prostate volume
CLSS: Core Lower Urinary Tract Symptoms score
AUC: Area under the curve
MCC: Maximal cystometric capacity
IPP: Intravesical prostatic protrusion
BC: Bladder capacity
PNI: Prognosis Nutrition Index
SII: Systemic Immune Inflammation Index
NLR: Neutrophil to Lymphocyte Ratio
PLR: Platelet to Lymphocyte Ratio
DUASQ: DUA Symptom Questionnaire
LUTD: Lower urinary tract dysfunction
BCa: Bladder cancer
BPH: Benign prostatic hyperplasia
CIC: Clean Intermittent Catheterization
DO: Detrusor overactivity
IC: Interstitial cystitis
IPSS: International Prostate Symptom Score
IPSS-QOL: International Prostate Symptom Score Quality of Life
LUTD: Lower urinary tract dysfunction
MS: Multiple sclerosis
PCa: Prostate cancer
PIP: Projected isovolumetric pressure
PSA: Prostate specific antigen
PSAD: Prostate-specific antigen density
PVR: Post-void residual
US: Ultrasound
UTI: Urinary tract infection
DWT: Detrusor wall thickness
VV: Voided volume
